# Supplementary material for: Gene Cloning and Characterization of Transcription Factor FtNAC10 in Tartary Buckwheat (Fagopyrum tataricum (L.) Gaertn.)
Source: Int J Mol Sci. 2023 Nov 14;24(22):16317. doi: 10.3390/ijms242216317 (PMC10671190; doi:10.3390/ijms242216317)
Supplement: Supplementary file 1 [file ijms-24-16317-s001.zip › ijms-2626431-supplementary.pdf]

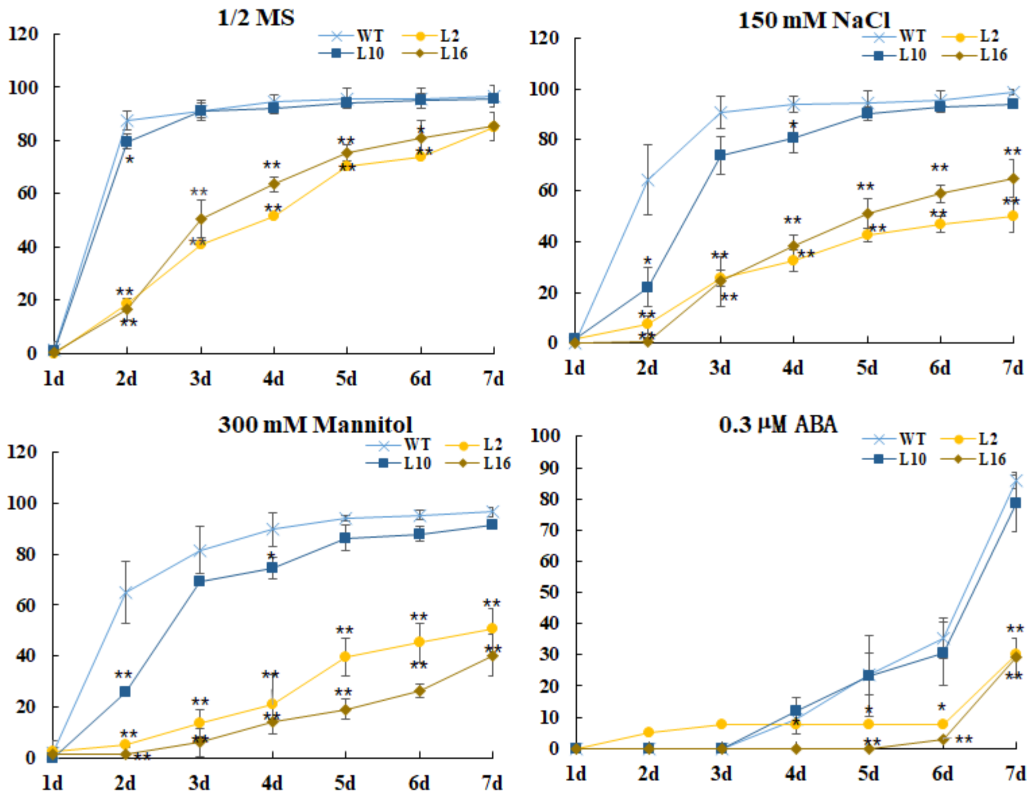

Figure S1: Germination statistical analysis of FtNAC10 transgenic *A. thaliana* lines on stress plate. The stress treatments were 150 mM NaCl, 300 mM Mannitol and 0.3 μM abscisic acid (ABA), respectively. Significant differences in germination rate between different transgenic lines were analyzed at level of 0.05 ($p < 0.05$) and compared with WT.
